# Use of a microsurgical vascular clip system for temporary bilateral occlusion of the four main uterine vessels for laparoscopic enucleation of very large intramural uterine fibroids

**DOI:** 10.1007/s00404-022-06675-1

**Published:** 2022-07-27

**Authors:** Shadi Younes, Marc Radosa, Achim Schneider, Julia Radosa, Alexey Eichenwald, Christiane Weisgerber, Bahriye Aktas

**Affiliations:** 1 Department of Obstetrics and Gynecology, University Hospital of Leipzig, Leipzig, Germany; 2 Department of Obstetrics and Gynecology, Klinikum Bremen Nord, Bremen, Germany; 3 Institute for Cytology and Dysplasia, MVZ im Fürstenberg-Karree, Berlin, Germany; 4 Department of Obstetrics and Gynecology, Saarland University Hospital, Homburg, Germany

**Keywords:** Large intramural myoma, Laparoscopic myomectomy, Yasargil^®^ clip, Temporary clipping of uterine vessels, Blood loss

## Abstract

**Objectives:**

The goal of this study was to examine the safety, feasibility, and effectiveness of the use of a microsurgical temporary vascular clip system to facilitate the laparoscopic enucleation of very large intramural uterine fibroids.

**Methods:**

In this retrospective study, the surgical outcomes of 26 patients who underwent laparoscopic myomectomy with temporary uterine vessel clipping for very large (the largest measured diameter ≥ 9 cm) symptomatic intramural uterine fibroids in two tertiary referral hospitals between September 2017 and March 2020 were examined. Titan-made vascular clips (YASARGIL^®^ Aneurysm Clip System) were used to temporarily occlude the bilateral uterine arteries and utero-ovarian vessels. Main outcomes included operating time, blood loss, number of leiomyomas and weight, conversion rate, intra- and postoperative complication rates, and length of hospital stay.

**Results:**

Twenty six patients were included. Dominant intramural uterine fibroid diameters were 9–22 cm. The general characteristics of the patients were similar. The mean surgery duration and intraoperative blood loss were 175.3 ± 32.7 (range 120–250) min and 241.1 ± 103 (range 100–450) ml, respectively. The median postoperative drop in hemoglobin was 0.89 ± 0.75 g/dL. No patient required blood transfusion. No procedure was converted to laparotomy. No major intra- or postoperative complication occurred.

**Conclusions:**

Laparoscopic myomectomy for very large intramural uterine fibroids can be performed safely and effectively, with less intraoperative blood loss, using vascular clips for temporary clamping of the bilateral uterine vessels.

## What does this study add to the clinical work

**Table:** 

Due to the temporary bilateral clamping of the main uterine vessels with special vessel clips prior to laparoscopic myomectomie, the size of the uterine leiomyoma is no longer the decisive indicator for the contraindication of a laparscopic myomectomy. Further studies are needed to examine the positive effects of this technique on future pregnancy outcomes.	

## Introduction

Increasing numbers of women in higher income countries are attempting to delay childbearing to older ages, when the incidence of uterine fibroids is increased; uterus-preserving myomectomy is thus an important alternative for this patient group [1, 2]. As with other gynecological benign diseases, individualized treatment approaches have increasingly gained in demand from patients in the therapy of fibroids over the past few years [3]. In the last three decades, the laparoscopic route has been established as the approach of choice for surgical treatment of uterine fibroids. Laparoscopic fibroid enucleation is associated with a low morbidity rate and high patient satisfaction levels [4]. With technical advances in instrumentation and techniques, most fibroids can be resected using minimally invasive techniques. However, laparoscopic myomectomy for an intramural uterine fibroid can be difficult and challenging, even for an experienced surgeon [5, 6], and especially when the fibroid is large. The well-vascularized myometrium can pose a technical challenge in endoscopic fibroid enucleation; diffuse bleeding may lead to significant intraoperative hemorrhage and require extensive use of bipolar or monopolar diathermy to achieve adequate hemostasis, which in turn might lead to postoperative uterine wall necrosis with inadequate wound healing and a potential risk of uterine rupture during subsequent pregnancy [7]. Bleeding is not the only problem encountered during the surgical treatment of large uterine myomas; the need for intracorporal reconstruction of the uterine wall with its excessive cut edges due to expansion of the myometrial layer by a large intramural fibroid also poses another challenge. This procedure can result in excessive blood loss requiring transfusion, prolongation of the operating time, and postoperative complications requiring prolonged hospital stays [2, 8].

To address this clinical challenge, we have temporarily interrupted the uterine blood supply by applying microsurgical vascular clips (Yasargil^®^ vascular clip system; Aesculap, Tuttlingen, Germany) to the uterine arteries and utero-ovarian vessel arcade to optimally minimize intraoperative bleeding during the endoscopic enucleation of very large intramural uterine fibroids. Yasargil^®^ vascular clips were originally used in the treatment of intracranial aneurysms [9]. In gynecological surgery, they have been used to treat vascular injuries during laparoscopic procedures [10] and for temporary clipping of uterine arteries during laparoscopic myomectomy for normally sized uterine fibroids [11–13]. In this study, we examined the feasibility, safety, and efficacy of temporary bilateral occlusion of the uterine arteries and utero-ovarian vessels with Yasargil^®^ clips during laparoscopic myomectomy in women with very large symptomatic intramural uterine fibroids in terms of blood loss and related perioperative outcomes.

## Materials and methods

### Patients and setting

**Fig. 1:**
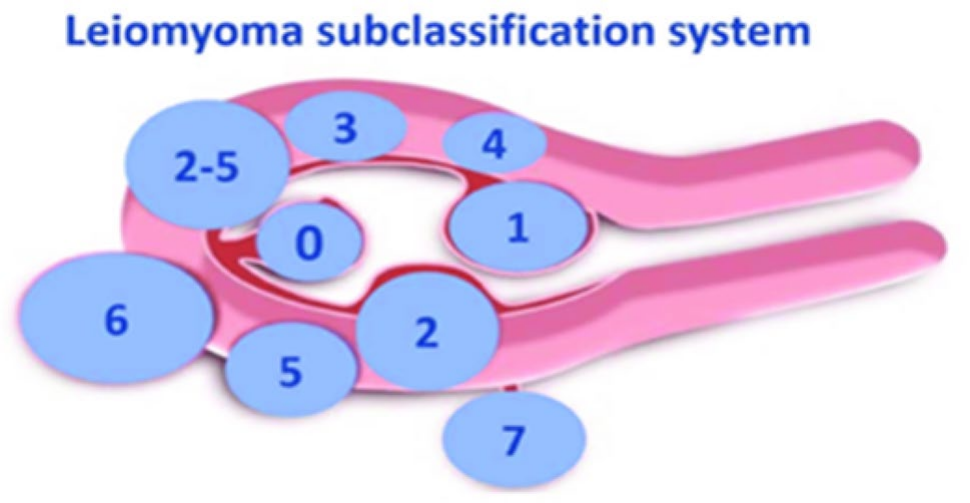
FIGO leiomyoma subclassification system. Reprinted from Ref. [15]. Copyright 2011. with permission, from Ref. [12]. ((Licensed Content Publisher: John Wiley and Sons—Licensed Content Autor: Malcom G. Munro. Granted Licensed Number: 52129990134061))

We included patients with symptomatic large intramural uterine fibroids who had undergone laparoscopic myomectomy with the use of microsurgical clips for temporary bilateral occlusion of the uterine vessels between September 2017 and March 2020 at two gynecological endoscopic referral centers: the Department of Gynecology of the University Hospital Leipzig (Germany) and the academic teaching hospital Agaplesion Diakonie Kliniken (Kassel, Germany). Women with at least one very large (diameter ≥ 9 cm) intramural leiomyoma (Munro types 3–6 [14]) (Fig. 1) were eligible for study inclusion. Patients with submucosal or subserosal uterine leiomyomas (Munro types 0–2 and 7) were excluded. Preoperatively, all patients provided informed consent to the temporary use of Yasargil® clips on the uterine vessels to facilitate laparoscopic myomectomy.

### Data collection

A total of 26 patients were eligible. Paper and electronic medical records of all patients were reviewed. Data on patients’ age, body mass index, parity, and previous history of pelvic surgery were collected. We also recorded leiomyoma number, maximum diameter, and location (submucosal, intramural, subserosal, pedunculated) determined by transabdominal/transvaginal sonographic and magnetic resonance imaging (MRI). The following intraoperative data were recorded: operative time (from the first skin incision to the end of skin wound closure); clip application time (from the initial opening of the retroperitoneal space on one side to the occlusion of the last uterine vessels on the opposite side including the time which needed to occlude the utero-ovarian vessels); clip application duration; number, locations, and sizes of all resected leiomyomas; total weight of all leiomyomas removed; estimated blood loss (blood volume in the suction canister at the end of the operation, excluding irrigation fluid); need for blood transfusion; intraoperative complications (e.g., excessive blood loss, pelvic organ injury); and changes of operating procedure or technique. Finally, data on postoperative complications [e.g., wound healing disorders, signs of infection (fever, leukocytosis, C-reactive protein increase), acute urinary retention, need for antibiotics, need for subsequent exploratory laparotomy], hemoglobin level on postoperative day 3, and length of hospital stay were extracted.

### Assessment

The size of each patient’s dominant leiomyoma was determined preoperatively by transabdominal sonography and/or MRI. Doppler examination of the uterine arteries was performed pre- and postoperatively by the same surgeon to ensure that clipping had no embolic effect on the uterine vessels. The resistance index (RI) was measured bilaterally to the ascending branch at the level of the internal cervical os, and the right and left RI values were averaged. Due to technical difficulties and the anatomical distortions caused by very large uterine fibroids, the utero-ovarian vessels were not assessed preoperatively.

### Surgical technique

Two highly qualified endoscopic surgeons with at least 5-year experience in minimally invasive gynecologic surgery performed all surgeries using the same technique. No preoperative treatment with a gonadotropin-releasing hormone agonist or ulipristal acetate (UPA) was administered, and no local intraoperative injection of a vasoconstrictive nore any application of antifibrinolytic drugs were performed.

The procedure was carried out under general anesthesia with the patient in the dorsal lithotomy position. A Verres needle was introduced three finger widths supraumbilically at the midline, and carbon dioxide was passed through it for intra‐abdominal inflation to a pressure of 12 mm Hg. We use low-pressure laparoscopy to minimize postoperative pain and avoid subcutaneous emphysema caused by prolonged operation times [15]. Upon the establishment of pneumoperitoneum, a 10‐mm trocar was inserted for the video laparoscope supraumbilically. Two 5-mm ports were inserted in the left and right lower abdominal regions, and a 12-mm assistant port was inserted midway between the umbilicus und symphysis.

The patient was then placed in the Trendelenburg position (~ 25°). The peritoneal cavity was inspected and the uterine fibroid number, locations, and sizes were identified. The bilateral retroperitoneal spaces were opened, and the uterine artery was reached in > 90% of cases (depending on uterus mobility, location, size und shape of the large intramural uterine fibroide) by lateral access through peritoneal incision at the level of the pelvic brim lateral to the external iliac artery and infundibulopelvic ligament. The point at which the ureter crossed the external iliac artery was identified. Using blunt dissection, the medial pararectal space was developed and the first medial branch of the internal iliac artery (the uterine artery) was located by following the ureter caudally. Medial access was used in cases of very deep and wide intramural fibroids that reduced uterus mobility and free spaces in the true pelvis and retroperitoneal space, through peritoneal incision medial to the infundibulopelvic ligament and lateral to the ureter. With this access, wide exposure of the retroperitoneal space is not needed and the uterine arteries can be found easily by following the upper edge of the ureter to the point at which it crossed the uterine artery.

**Fig. 2:**
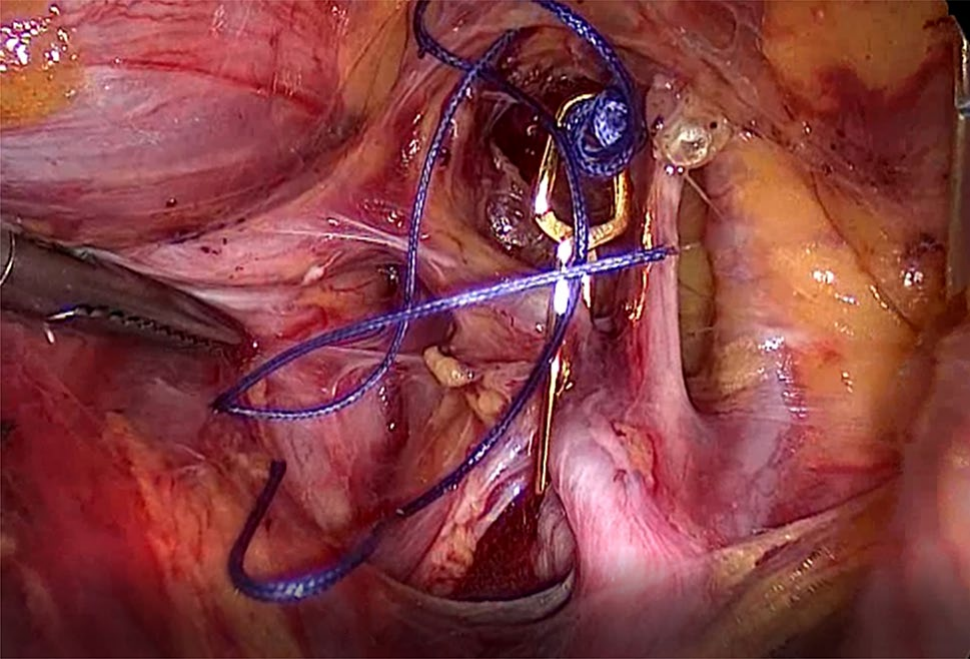
Transient occlusion of the uterine artery on the right side with straight 11 mm Yasargil^®^ clip (FE769K; Aesculap)

**Fig. 3:**
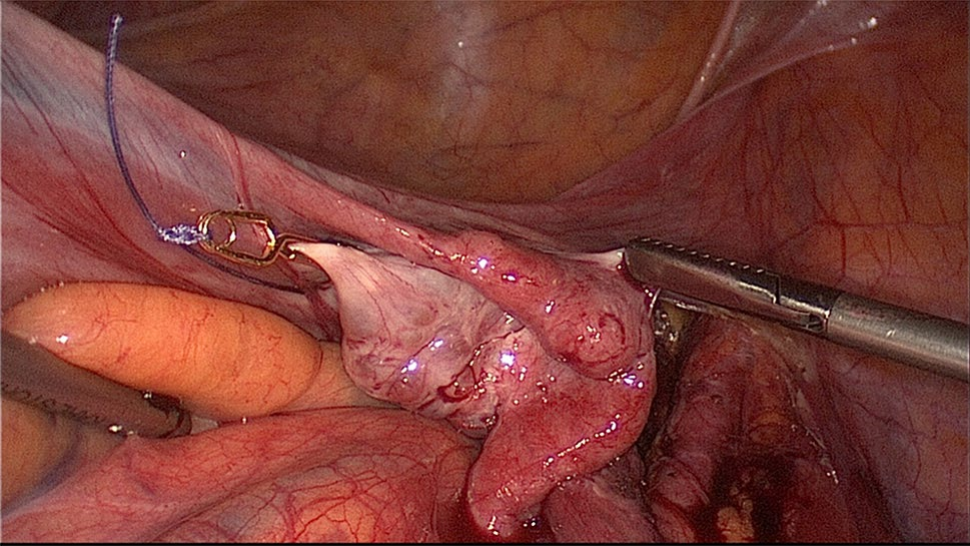
Transient occlusion of the uterine artery on the right side with curved 13.8 mm Yasargil^®^ clips (FE783K; Aesculap)

**Fig. 4:**
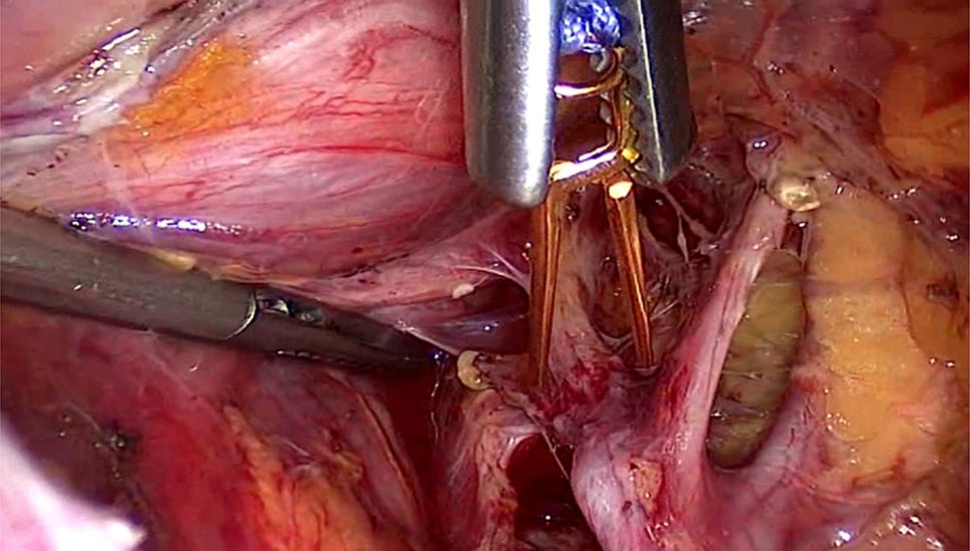
Vascular clip is inserted through the 10-mm trocar, is opened by an alligator forceps and placed over the uterine artery

**Fig. 5:**
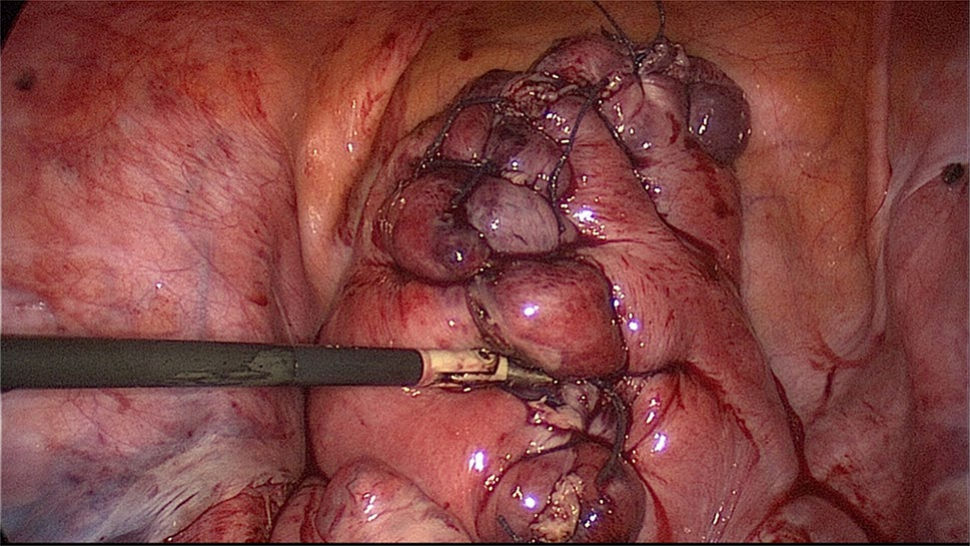
Reconstruction of the uterine wall with interrupted intracorporal double-layer sutures

Following uterine artery exposure and preparation, a 11-mm Yasargil^®^ clip (FE769K; Aesculap) (Fig. 2) was applied temporarily to each of the bilateral arteries in succession with a clamp force of 0.69 Newtons. The blood supply via the utero-ovarian vessel arcade was also occluded by applying curved 13.8 mm Yasargil clips (FE783K; Aesculap) (Fig. 3) to the bilateral ovarian ligaments. All four clips are marked with Vicryl sutures to facilitate identification toward the end of surgery. The titanium clips can be easily applied to and removed from the vessels using atraumatic alligator forceps (Fig. 4). The uterine serosa and myometrium were then incised, and the surface of each fibroid was exposed. Each fibroid was grasped with a 10-mm tenaculum forceps through the lower middle port and extracted from the surrounding myometrium by blunt dissection. The uterus was closed with interrupted intracorporal double-layer sutures (Vicryl 2, CTX, Ethicon) (Fig. 5).^1^ Taking advantage of the very large curved belly of the needle, an initial deep stitch to the ground of the myoma bed was used to close the deep uterine muscle layer without allowing dead space formation. The subsequent back-stitch was used to close the superficial uterine muscle layer with inner inversion of the uterine serosa to minimize adhesion surfaces at the hysterotomy scar. Following closure of the uterus, the Yasargil^®^ clips were removed, pulsation of the uterine arteries medial to the clipping positions was confirmed, and the blood circulation in the uterus was photo documented. The fibroids were morcellated using an electric morcellator and extracted through the midline trocar. At the end of the procedure, an intra-abdominal drain (French gauge 15) was placed for postoperative monitoring. We have published a video clip article on this technique [16].

### Postoperative assessment and care

Histopathological evaluation of the excised fibroids was performed. All patients received intravenous metamizol (Novalgin^®^) and/or acetaminophen (Perfalgan^®^) according to the standard protocol for postoperative pain control. Patients were discharged home on the day that they achieved immediate postoperative milestones (ambulation, pain control, oral liquid tolerance, and successful voiding). Discharge medications included oral ibuprofen and acetaminophen. All patients attended standard follow-up visits at 2 weeks and 3 months postoperatively. Revascularization of the uterus was evaluated using Doppler ultrasound.

### Statistical analysis

The Statistical Package for the Social Sciences (SPSS 22.0 Inc., Chicago IL, USA) was used for statistical analysis. Continuous variables are reported as means and standard deviations, and discrete variables are reported as percentages of the total group. Box and whisker plots were created for the preoperative hemoglobin level, number and locations of removed leiomyomas, leiomyoma weight, postoperative hemoglobin drop, and intraoperative blood loss. Differences were considered significant at *p* < 0.05.

## Results

The study sample comprised 26 patients with a mean age at the time of surgery of 32.3 (range 25–42) years. Laparoscopic myomectomy was performed to treat primary and secondary infertility associated with fibroids (*n* = 18), bleeding disorders (*n* = 15), dysmenorrhea (*n* = 7), pelvic pain (*n* = 9), dyspareunia (*n* = 3), and urinary symptoms (*n* = 1; Table 1).

**Table 1:** Characteristics of patients with large intramural uterine fibroids

The basic characteristics of patients with huge intramural uterine fibroids	
	Mean (SD)
Age	32.38 (5.02)
Body mass index (kg/m^2^)	23.76 (5.58)
Preoperative hemoglobin (g/dl)	10.84 (1.3)
Pregnancy history (*n* (%))	
Nullipara	14 (53.8%)
Multipara	12 (46.2%)
Previous abdominal or pelvic surgery (*n* (%))	6 (23%)
Main indication for myomectomy (*n* (%))	
Primary or secondary infertility	18 (69.2%)
Bleeding disorder	15 (57.6%)
Dysmenorrhea	7 (26.9%)
Pelvic pain	9 (34.6%)
Dyspareunia and	3 (11.5%)
Urinary symptoms	1 (3.8%)

Data are expressed as mean ± standard deviation or (*n* (%)); (*n* = 26)

A mean of 2.9 (range 1–19) leiomyomas per patient were removed. The mean dominant leiomyoma size was 12.3 (range 9–22) cm. Dominant leiomyomas were anterior in 5 patients, posterior in 9, fundal in 10, and intraligamental in 2 patients (Table 2).

**Table 2:** Characteristics of resected uterine fibroids

Characteristics of the resected uterine fibroids	
	Mean ± SD (range)
Number of myomas (resected)	2.9 ± 3.49 (1–19)
Size of dominant myoma (mm) (sonography/MRI)	12.38 ± 4.07 (9–22)
Total weight of myomas (g)	848.2 ± 268.19 (505–1520)
	(*n* (%))
Site of operated myoma	
Intramural	26 (100%)
Subserous	8 (30%)
Pedunculated	2 (7.6%)
Location of dominant myoma	
Anterior	5 (19.2%)
Posterior	9 (34.6%)
Fundus	10 (38.4%)
Broad ligament	2 (7.6%)

Data are expressed as mean ± standard deviation, range or (*n* (%)); (*n* = 26)

The mean clip application time was 21.80 ± 6.22 (range 12–35) min and the mean clip application duration was 106 ± 20.19 (range 60–130) min. The mean intraoperative blood loss was 241.1 ± 103.0 (range 100–450) ml, the mean operative time was 175.3 ± 32.7 (range 120–250) min, and the mean hospital stay was 3.15 ± 0.8 (range 2–6) days. No conversion to laparotomy or surgical technique-related complication occurred. In addition, no severe intraoperative complication (e.g., vascular, ureter, bladder, or bowel injury) occurred. In one patient, we could not apply a clip to the right uterine artery because of fibrotic alteration of the retroperitoneal tissue with endometriosis infiltration. However, we achieved total resection of the leiomyoma with no complication, with 150 ml estimated blood loss. Pathological examination revealed nonmalignant uterine leiomyomas in all cases.

The largest intramural leiomyoma removed was 22 cm in diameter; it was located in the anterior-fundal uterine wall. In this patient, the estimated intraoperative blood loss was 250 ml and the operative time was 190 min; no intraoperative complication was documented. The heaviest leiomyoma removed was 1520 g; we removed 19 fibroids, more than half of which were intramural, from this patient. This case had the highest blood loss (450 ml) and longest operative time (480 min), but the postoperative hemoglobin level was 9.3 g/dl and no blood transfusion were required. Postoperatively, the patient developed a massive upper body and cervicofacial subcutaneous emphysema, which resolved spontaneously after 24 h. Postoperative ultrasound examination revealed a non-symptomatic intramural hematoma measuring 2 × 3 cm in one patient with a normal clinical course, which had disappeared completely at the 3-month follow-up examination (Table 3).

**Table 3:** Intra- and postoperative outcomes

Characteristics of the surgery and the postoperative outcomes	
	Mean ± SD (range)
Operating time (min)	175.3 ± 32.7 (120–250)
Time of Clips application on uterine vessels (min)	21.80 ± 6.22 (12–35)
Application time of Clips on uterine vessels (min)	106 ± 20.19 (60–130)
intraoperative blood loss (ml)	241.1 ± 103.0 (100–450)
Postoperative hemoglobin (g/dL)	9.94 ± 1.21 (7.5–13)
Hemoglobin drop (g/dL)	0.89 ± 0.75 (0–2.8)
Length of hospital stay (days)	3.15 ± 0.8 (2–6)
	(*n* (%))
Blood transfusion	0
Intraoperative complications	1 (3.8%)^a^
Conversions to laparotomy	0
Postoperative complications	3 (11.5%)
Urinary retention	1 (3.8%)
Febrile urinary tract infection	1 (3.8%)
Incisional hematoma	1 (3.8%)

Data are expressed as mean ± standard deviation, range or *n* (%); (*n* = 26))

^a^Cervicofacial subcutaneous emphysema that resolved spontaneously after 24 h

The mean pre- and postoperative RIs were 0.82 (range 0.67–0.89) and 0.80 (range 0.65–0.88), respectively; these values did not differ significantly (Table 4). Also an unsuspicious vascularization Doppler patterns of both ovarian postoperatively was documented. The mean postoperative hemoglobin drop was 0.89 ± 0.75 g/dl.

**Table 4:** Uterine artery resistance indices

	Before surgery	3d after surgery	*p* value
	Mean (min–max)		
Uterine artery resistance index (RI)	0.82 (0.67–0.89)	0.80 (0.65–0.88)	ns

Differences considered significant at *p* < 0.05

## Discussion

Since Semm [17] first introduced laparoscopic myomectomy with vaginal extraction of uterine myomas, numerous studies on its feasibility and safety have been published [18–20]. The procedure has been associated with a reduced postoperative hemoglobin drop and less blood loss [21, 22], as well as less morbidity and shorter hospital stay with less postoperative pain [23]. However, few articles describe the use and safety of laparoscopy for the removal of very large intramural uterine fibroids. The available data indicate that the use of laparoscopic myomectomy for this purpose entails more intraoperative blood loss, difficulties in enucleating the fibroids, a high rate of conversion to laparotomy, inadequate endoscopic suturing leading to the risk of uterine rupture during pregnancy, longer operation times, and more surgery-related complications [24–26]. The permanent or temporary reduction of the uterine blood supply prior to myomectomy was developed to overcome this problem, and various techniques have been used successfully in minimally invasive uterine fibroid procedures [27, 28]. In a randomized controlled trial, Vercellino et al. [13] demonstrated the effectiveness of temporary laparoscopic clipping of the uterine arteries before myomectomy in terms of reduced blood loss with no related postoperative side effect on the uterine vessels. In the present study, we focused on very large intramural uterine fibroids, which are the most difficult and complex cases. Our sample is among the largest of its type reported in the literature.

Saccardi et al. [6] showed that leiomyoma size and type were good predictors of blood loss, with leiomyomas of 8–12 cm associated with significantly increased blood loss (almost twice as high as in smaller leiomyomas) and the removal of intramural myomas larger than 12 cm causing a median of 450 ml blood loss. The technique used in the present study reduced intraoperative bleeding to a mean of 241.1 ml; 450 ml blood loss occurred in one patient in which we resected 19 leiomyomas, the largest of which was intramural and 10 cm in diameter. The reduction of intraoperative blood loss was also confirmed by the detection of a significantly lower mean postoperative hemoglobin drop than reported in the literature [29].

In this study we used Yasargil^®^ clips, which Vercellino et al. [13] used in their randomized trial. Temporary Yasargil^®^ clips, made of titanium, are employed in neurosurgery, vascular surgery, and increasingly in gynecological surgery [12, 13, 16]. Their use with the controlled exertion of pressure recommended by the manufacturer (0.69 newtons for FE769K and FE783K) reduces the risk of vascular wall damage [10, 11, 30]. We chose to use this small temporary clip system in this study, because the clips are easy to introduce intraabdominally via the laparoscopic route and because their small size is advantageous when applying them to the uterine vessels on a pelvic side wall near a large myoma. Clip application to the uterine vessels enabled us to provide adequate hemostasis in the surgical field during myoma cleavage and after enucleation, which allowed more time to close the hysterotomy and to apply the sufficient sutures (i.e., one, two or more layers) without experiencing time stress because of excessive bleeding. It also allowed us to avoid unnecessary and damaging coagulation of the myometrium, which improves uterine healing and reducies the risk of scarring necrosis and consequently uterine rupture [7, 31].

Although intraoperative bleeding and the intramural uterine fibroid recurrence rate can be reduced markedly by permanent uterine artery occlusion before laparoscopic myomectomy and postoperative involution of the small myoma (necrobiosis) [27, 28], these procedures can have negative effects on future fertility [29]. Temporary occlusion of the uterine vessels, as performed in this study, guarantees complete revascularization of the uterine muscles and endometrium directly after clip removal, which is easily proven macroscopically by the observation of uterus re-gaining normal color or postoperative Doppler examination; in this study, the lack of change in the RI confirmed that clipping had no negative effect on the pulsatility of the uterine arteries. The preservation of uterine vascularization is favorable for a normal healing process.

Clip application in our technique increased duration of the surgery. In 85% of our patients, especially those with good uterine mobility, clip application could be achieved in about 20 min. In four patients with topographically deep fibroids (two intraligamental, one retrocervical, one intramural), clip application took about 30–35 min longer. However, this prolongation had no negative effect on the overall mean operative time relative to that required for standard laparoscopic myomectomy for similarly sized leiomyomas (153.3 ± 40 min) [5], during which surgeons may need more time to achieve adequate hemostasis to control excessive bleeding. To our experience, for a surgeon who routinely performs laparoscopic myomectomy, identifying and occluding the uterine artery is a technical step that requires little learning curve to be performed in a timely and safely manner. In addition, we took also more time for myometrial reconstruction, because we used interrupted intracorporal double-layer sutures, which are of similar quality as sutures used in open surgery. This technique provides more stability and safety for future pregnancies via better healing of the hysterotomy scar and a reduced potential for uterine rupture during pregnancy [7, 31].

The uterine artery ligation time is a controversial issue related to this study. Wang et al. [2] used an average occlusion period of approximately 2 h for laparoscopic myomectomy, with no related complications occurring. In our experience, we have not found that the uterine artery occlusion period represents a considerable problem due to collateral anastomosis (vaginal ascending arteries, vesico-cervical anastomosis, Sampson‘s artery, small branches from the ligament latum), which are small branches but functionally very important during occlusion time. In the present study, the transient ligation period (maximum, 130 min) was harmless to the myometrium and caused no related clinical complication.

The potential need to convert a laparoscopic procedure to laparotomy is an important concern. Limited mobility and accessibility, and the unusual and poor approach angle of laparoscopic instruments, impede the rapid management of acute complications, such as excessive hemorrhage, which is the most decisive factor forcing conversion. Dubuisson et al. [19] showed that fibroid size is a primary factor in such decisions. In another large-scale study including 444 laparoscopic myomectomies, Saccardi et al. [6] showed that the incidence rate of conversion to laparotomy was 25% in cases with intramural uterine fibroids more than 12 cm in diameter. In our current study, we were not forced to convert to laparotomy in any patient.

In addition, the median hospital stay in the present study was comparable to those reported for laparoscopic myomectomy performed to treat uterine fibroids in premenopausal women [32] and to treat large uterine myomas [33].

Another important issue to recognize is related to the locations of the trocars and smooth handling of the instruments without obstruction by a large uterus, especially when uterine artery clipping is desired. To minimize problems, we recommend placement of the main optic trocar midway between the xiphoid process and umbilicus, the central accessory trocar between the umbilicus and symphysis, and the remaining two accessory trocars lateral to the umbilicus. This modification provides more intraperitoneal space for manipulation with minimal intervention of a large uterus.

In the present study, we did not use pre-medication or vasoconstricting agents pre- or intraoperatively to reduce surgery-related bleeding. The local administration of diluted vasopressin to the uterus at the beginning of the procedure (usually by intramural injection before uterine incision) is used to induce vasoconstrictive effects on the small vessels surrounding a leiomyoma. This technique is considered to be safe and effective for the control of regional blood flow from the uterine vessels to reduce hemorrhage [34]. However, as severe complications of vasopressin use, including pulmonary edema, severe hypotension, and bradycardia with eventual cardiac arrest, have been reported, increasing numbers of surgeons are abandoning this technique [35, 36]. Regarding the use of premedication to increase the safety and effectiveness of surgery for large uterine fibroids, pretreatment with a selective progesterone–receptor modulator, resulted in symptomatic improvement and the reduction of fibroid size in a large cohort of women of all ethnicities and ages [37]. In the same study, UPA treatment was shown to induce amenorrhea, reduce heavy menses, and improve quality of life in women with huge myomas who were not seeking parenthood or undergo surgery, but 25% of fibroids failed to respond to them. Furthermore, we have noted in our daily practice a greater likelihood of progressive myoma growth immediately after UPA treatment termination. From a surgical point of view, laparoscopic myomectomy is subjectively and technically more difficult after UPA pretreatment, which affects the morphological structure of the myometrium by shrinking the myometrial fibers and stroma, thereby softening the myometrial layers [38]. This effect can hamper identification of the correct dissection plane between the myoma and the myoma pseudocapsule, making dissection of the myoma much more difficult and lengthier and in turn resulting in more intraoperative blood loss.

We acknowledge several limitations of this study. First, the study was retrospective, had a comparatively small study group size and lacked a comparison group of women treated by the same surgeons. To further evaluate the use of microsurgical vascular clip systems with regard to a potential benefit in the endoscopic therapy of fibroids, a prospective randomized study is required which also addresses aspects, such as patient satisfaction or cost efficiency. Second, the performance of laparoscopic myomectomy for very large intramural leiomyomas requires more technical skill compared to other laparoscopic myomectomies procedures, and clip application to the uterine arteries in such patients is a specific minimally invasive surgical skill requiring expertise in the retroperitoneal pelvic anatomy. Third, we did not examine the effects of our technique on future pregnancies or the fertility rate, course of pregnancy, or risk of uterine rupture. We intend to examine these long-term outcomes in a follow-up study.

In conclusion, laparoscopic myomectomy for very large intramural uterine leiomyomas, can be performed safely and more effectively, with less intraoperative blood loss, via temporary bilateral clamping of the uterine vessels with special vascular clips. Despite the additional time required for clip application, the overall operating time was not increased. Further studies are needed to examine the effects of this technique on future pregnancy outcomes.

## Footnotes

1 The author affirm that human research participants provided informed consent for publication of the images in Figs. 2, 3, 4, 5.
